# Maternal and female fetal testosterone levels are associated with maternal age and gestational weight gain

**DOI:** 10.1530/EJE-17-0207

**Published:** 2017-07-11

**Authors:** Theodora Kunovac Kallak, Charlotte Hellgren, Alkistis Skalkidou, Lotta Sandelin-Francke, Kumari Ubhayasekhera, Jonas Bergquist, Ove Axelsson, Erika Comasco, Rebecca E Campbell, Inger Sundström Poromaa

**Affiliations:** 1 Departments of Women’s and Children’s Health; 2 Chemistry – BMCAnalytical Chemistry and Neurochemistry, Uppsala University, Uppsala, Sweden; 3 Centre for Clinical Research SörmlandUppsala University, Eskilstuna, Sweden; 4 Department of NeuroscienceUppsala University, Uppsala, Sweden; 5 Centre for NeuroendocrinologyDepartment of Physiology, University of Otago School of Medical Sciences, Dunedin, New Zealand

## Abstract

**Objective:**

Prenatal androgen exposure has been suggested to play a role in polycystic ovary syndrome. Given the limited information on what maternal characteristics influence maternal testosterone levels, and the even less explored routes by which female fetus androgen exposure would occur, the aim of this study was to investigate the impact of maternal age, BMI, weight gain, depressed mood and aromatase SNPs on testosterone levels in maternal serum and amniotic fluid of female fetuses.

**Methods:**

Blood samples from pregnant women (*n* = 216) obtained in gestational weeks 35–39, and pre-labor amniotic fluid samples from female fetuses (*n* = 56), taken at planned Caesarean section or in conjunction with amniotomy for induction of labor, were analyzed. Maternal serum testosterone and amniotic fluid testosterone and cortisol were measured by tandem mass spectrometry.

**Results:**

Multiparity (β = −0.28, *P* < 0.001), self-rated depression (β = 0.26, *P* < 0.001) and weight gain (β = 0.18, *P* < 0.05) were independent explanatory factors for the maternal total testosterone levels. Maternal age (β = −0.34, *P* < 0.001), weight gain (β = 0.19, *P* < 0.05) and amniotic fluid cortisol levels (β = 0.44, *P* < 0.001) were independent explanatory factors of amniotic fluid testosterone in female fetuses, explaining 64.3% of the variability in amniotic fluid testosterone.

**Wider implications of the findings:**

Young maternal age and excessive maternal weight gain may increase the prenatal androgen exposure of female fetuses. Further studies are needed to explore this finding.

## Introduction

The impact of prenatal testosterone exposure has been a focus of interest in the last decades. Testosterone plays an important role in the organization and sexual differentiation of the brain during early fetal development, and exposure to high levels of testosterone during critical periods of fetal life promotes behavioral masculinization in a variety of mammals (1). Most of the evidence concerning human behavior comes from studies on women with congenital adrenal hyperplasia (CAH), who during childhood and adolescence display increased male-typical toy-, play- and playmate preferences (1).

However, increased levels of testosterone during fetal life are also suggested as one of the potential causes of PCOS development. Sheep (2), monkeys (3), rats (4) and mice (5, 6) prenatally treated with testosterone or dihydro-testosterone exhibit ovarian and endocrine traits similar to women with PCOS, such as LH hypersecretion, enlarged polyfollicular ovaries and functional hyperandrogenism. Yet, little is known about how such androgen exposure would occur in humans.

Maternal serum testosterone concentrations increase by 70% during pregnancy (7) and are increased to an even greater degree in women with PCOS (8, 9) and preeclampsia (10). In addition, young maternal age is associated with higher testosterone levels in pregnancy (11, 12, 13). However, the relationship between maternal androgen levels and maternal BMI, weight gain and depression are still less well explored (14).

Generally, maternal testosterone levels are not correlated with the testosterone concentration in the fetal circulation, at least not in conjunction with delivery when the fetal circulation becomes accessible for assessment (15, 16, 17). Presumably, the presence of the aromatase enzyme in the placenta, converting androgens into estrogens, protects the female fetus from direct transfer of maternal testosterone (18). The aromatase enzyme, which is encoded by a gene member of the cytochrome P450 family enzyme, subfamily A, polypeptide 1 (*CYP19A1*), is about 130 kb long, and located on chromosome 15q21. CYP19A1 tissue-specific expression is regulated by the 5′ flanking region of the gene, which includes multiple tissue-specific promoters (19). Several polymorphisms have been found in the aromatase gene; associations, though sparse, have been found with sex hormones, hyperandrogenism as well as obesity. Among these, the single nucleotide polymorphism (SNP) rs700518 (T > C), located in a coding region of the *CYP19A1* gene has been consistently associated with inter-individual differences in estradiol serum levels in men (20, 21, 22), but also with pre-eclampsia (23) and changes in body composition during treatment with aromatase inhibitors (24).

Testosterone levels in the fetus may be assessed at delivery, via the umbilical cord or during pregnancy via the amniotic fluid. From the second trimester and beyond the main source of testosterone into amniotic fluid is via urine from the fetus (25). Mean testosterone levels in the amniotic fluid of male fetuses are significantly higher than those in the amniotic fluid of female fetuses at all stages of gestation (26). It is assumed that the testosterone detected in amniotic fluid of female fetuses is primarily of adrenal origin, where it is partly regulated by adrenocorticotropic-releasing hormone (ACTH) (27).

Given the limited information on what maternal characteristics influence maternal testosterone levels, and the even less explored routes by which female fetus androgen exposure would occur, the aim of this study was to investigate the impact of maternal age, BMI, weight gain, depressed mood and aromatase SNPs on testosterone levels in maternal serum and in amniotic fluid of female fetuses.

## Subjects and methods

### Subjects

Both maternal blood samples and amniotic fluid samples were derived from the ‘Biology, Affect, Stress, Imaging, and Cognition in pregnancy and the puerperium’ (BASIC) cohort. The BASIC study investigates biological correlates of mood and anxiety disorders during pregnancy and in the postpartum period (28, 29, 30, 31). All pregnant women in Uppsala County, Sweden are invited to participate around their routine ultrasound examination in gestational week 17. Exclusion criteria for the BASIC study are (1) inability to adequately communicate in Swedish, (2) confidentially kept personal data, (3) ultrasound findings of severe fetal anomalies and (4) age less than 18 years. The women who participate (around 23% of the county births) fill out Web-based questionnaires, including the Swedish version of the Edinburgh Postnatal Depression Scale (EPDS) in gestational weeks 17 and 32. The study has been approved by the Regional Ethical Review Board in Uppsala.

#### Study population 1, maternal serum testosterone

BASIC participants with EPDS score ≥ 13 in gestational week 32, and a random sample of women with EPDS scores < 13 at gestational week 32 were invited for this sub-study to the BASIC project. The participants visited the research laboratory at the Department of Women’s and Children’s Health around gestational week 38 (range 35–39, according to the ultrasound-estimated date of delivery) between January 2010 and May 2013. The visits were scheduled between 08:00 h and 15:00 h, with the majority starting either at 09:00 h or at 13:00 h. A detailed medical history was completed, and obstetric outcomes were later retrieved from the medical records. Information on maternal BMI and maternal weight gain was obtained from the standardized antenatal medical records. Weight gain was defined as the difference between weight in gestational week 36 and weight at first antenatal booking, and this information was only available in 150 women. A venous blood sample was drawn, which was immediately centrifuged and stored in −70°C. Out of a total of 234 women who participated in the study, serum for this study was available from 216 women.

For the genetic analyses, non-Scandinavian ethnicity was an exclusion criterion. Genotyping was successfully completed in 201 women out of 215 women of Scandinavian ethnicity in study population 1.

#### Study population 2, amniotic fluid testosterone

By June 2015, 1129 amniotic fluid samples were available in the BASIC biobank. From these, we excluded samples from twin pregnancies (*n* = 20) and samples from male fetuses (*n* = 600), leaving 509 samples from female fetuses. In order to minimize the impact of labor stress (32), we included only samples taken at planned Caesarean section or at amniotomy for induction of labor (*n* = 56). Remaining samples were excluded because they had been obtained during labor. Caesarean section samples were taken in conjunction with the amniotomy, and all samples were immediately stored on dry ice and transferred to −70°C within one hour. Information on maternal and fetal characteristics was derived from BASIC questionnaires and medical records.

### Methods

#### Testosterone and cortisol assay

Steroid hormones were analyzed with Ultra-Performance Convergence Chromatography (UPC^2^; Waters ACQUITY UPC^2^, Milford, MA) coupled with tandem mass spectrometry (XEVO TQ-S, Milford, MA) (33). The analysis was performed with an Acquity UPC^2^ BEH column (150 mm 3.0 mm, 1.7 µm at 40°C (Waters, Milford, MA, USA)). The sample preparation (100 µL of serum or 500 µL of amniotic fluid) involves liquid extraction together with derivatization into methoxyamine prior to the analysis (33). The quantification of steroid hormones was done with multiple reactions monitoring (MRM) coupled with stable isotope dilution mass spectrometry. All data collected in centroid mode were obtained using Masslynx NT4.1 software (Waters Corp., Milford, MA USA).

Duplicate analyses of each sample were performed and the average values were reported (CV <6%). The linearity of the method was evaluated over a range of concentrations (0.17–104 nmol/L for testosterone and 0.14–1379 nmol/L for cortisol) and correlation coefficients (*R^2^*) were 0.998 and 0.999 for testosterone and cortisol, respectively. The limits of quantifications were 0.17 nmol/L for testosterone and 0.14 nmol/L for cortisol.

#### Analysis of serum sex hormone-binding globulin (SHBG) and albumin

SHBG was measured with immunometry electrochemiluminescense. The analyses were performed with a Roche Cobas601 and CobasElecsys SHBG reagent kits (Roche Diagnostics). Total coefficient of variation (CV) for SHBG was 3% at 38–109 nmol/L. Serum albumin was determined using BCP reagent from Abbott Diagnostics on an Architect 16000 (Abbott Laboratories). Total CV for serum albumin was 2% at 40.6–79.8 µmol/L. All analyses were performed at the accredited laboratory of Department of Clinical Chemistry, Uppsala University hospital. Two subjects were excluded due to insufficient sample volume. The Vermeulen method was used to estimate serum concentrations of bioavailable testosterone, by use of the Mazer spreadsheet (34), and calculations included SHBG, albumin and testosterone levels.

#### Analysis of aromatase single nucleotide polymorphisms

DNA was extracted from blood using the silica-based Kleargene DNA extraction method. Genotyping analyses of aromatase (*CYP19A1)* rs28757184, rs56658716, rs2236722, rs700518 and rs6493497 single nucleotide polymorphisms (SNPs) were performed using the Kbioscience Allele-Specific Polymorphism assay (KASP) based on competitive allele-specific PCR and bi-allelic scoring of the SNP. No-template control samples were included to enable the detection of contamination or non-specific amplification. Genotype and allele frequencies are reported in Supplementary Table 1 (see section on supplementary data given at the end of this article). Genotypes were in Hardy–Weinberg equilibrium. Linkage disequilibrium (D’ and *r*^2^) and potential haplotype blocks were estimated with the EM algorithm using SNP and Variation Suite 7 (GoldenHelix). The SNPs showed low linkage disequilibrium (*r*^2^) between each other, and no haplotypes blocks were detected.

### Statistical analysis

Sample size estimation for study population 1 indicated that 194 subjects would be required to detect a correlation between testosterone and BMI in the range of 0.2 (35), with a significance level of 0.05 and a power of 80%. No power analyses were made for study population 2, which can be regarded as a convenience sample.

Distributions of testosterone, bioavailable testosterone and cortisol did not follow the normal distribution (all positively skewed). Spearman correlations were used to assess the bivariate correlations, and group comparisons were made with the Kruskal–Wallis test or Mann–Whitney *U* test.

Multivariable linear regression analyses were performed to investigate the effect of age, BMI in early pregnancy, weight gain and depressive symptoms on total maternal testosterone levels. Natural logarithm transformed total testosterone levels were normally distributed and were used in the adjusted regression models to meet the assumption of randomly distributed residuals. Besides the variables of interest, inclusion of variables in the final models was based on significant associations in the bivariate analyses. Thus, the predictor variables for maternal testosterone included age, BMI, parity and EPDS scores in gestational week 32, with addition of gestational weight gain in a second model. Similarly, the predictor variables for amniotic fluid testosterone included maternal age, parity, BMI, gestational weight gain (dichotomized), assisted reproduction, gestational age and amniotic fluid cortisol levels. Gestational weight gain was dichotomized as above or below 1 standard deviation score (determined in the entire BASIC cohort), which equaled a weight gain > 18.9 kg at the end of pregnancy. Based on the literature, polycystic ovary syndrome was included in all models (8, 9). Finally, the linear regression model on amniotic fluid testosterone was performed with and without inclusion of cortisol levels. SPSS Statistics, version 23 for Windows was used for the analyses and *P* values <0.05 were considered statistically significant.

## Results

All women except one in each study population had Scandinavian ethnic background and all but one reported cohabiting with a partner. Further study population demographics are given in Table 1 along with steroid hormone levels.

**Table 1:** Background characteristics in mothers and hormone levels of the two study populations.

	**Study population I: maternal serum testosterone**		**Study population II: female fetus testosterone**	
**Characteristic**	***n***	**Mean/median/proportion**	***n***	**Mean/median/proportion**
Age, years	216	31.3 (4.5)	56	31.8 (5.5)
Primiparous subjects, *n* (%)	216	97 (43.5%)	56	21 (37.5%)
First trimester BMI (kg/m^2^)	210	24.5 (4.3)	56	25.3 (4.3)
Weight gain (kg)	150	12.9 (4.8)	55	11.0 (4.8)
Smoking during pregnancy, *n* (%)	216	8 (3.7%)	56	5 (8.9%)
Polycystic ovary syndrome, *n* (%)	215	5 (2.3%)	56	2 (3.6%)
Assisted reproduction	216	12 (5.6%)	56	7 (12.5%)
Depressed during pregnancy, *n*	216	59 (27.3%)	56	8 (14.3%)
Hypertensive disorders, *n* (%)	216	8 (3.6%)	56	4 (7.1%)
Total testosterone (nmol/L)	216	1.95 (1.40–2.74)	56	2.04 (1.12–4.18)
Bioavailable testosterone (nmol/L)	214	0.07 (0.04–0.12)		–
SHBG (nmol/L)	214	474 ± 131		
Cortisol (nmol/L)	216	163 (119–239)	56	457 (231–506)

Normally distributed data are displayed as mean (s.d.), and skewed data as median (interquartile range). Nominal data are given as *n* (%). Weight gain was recorded in the maternity health care records and was only available in 150 women.

### Maternal testosterone is influenced by parity, weight gain and depressive symptoms

**Figure 1:**
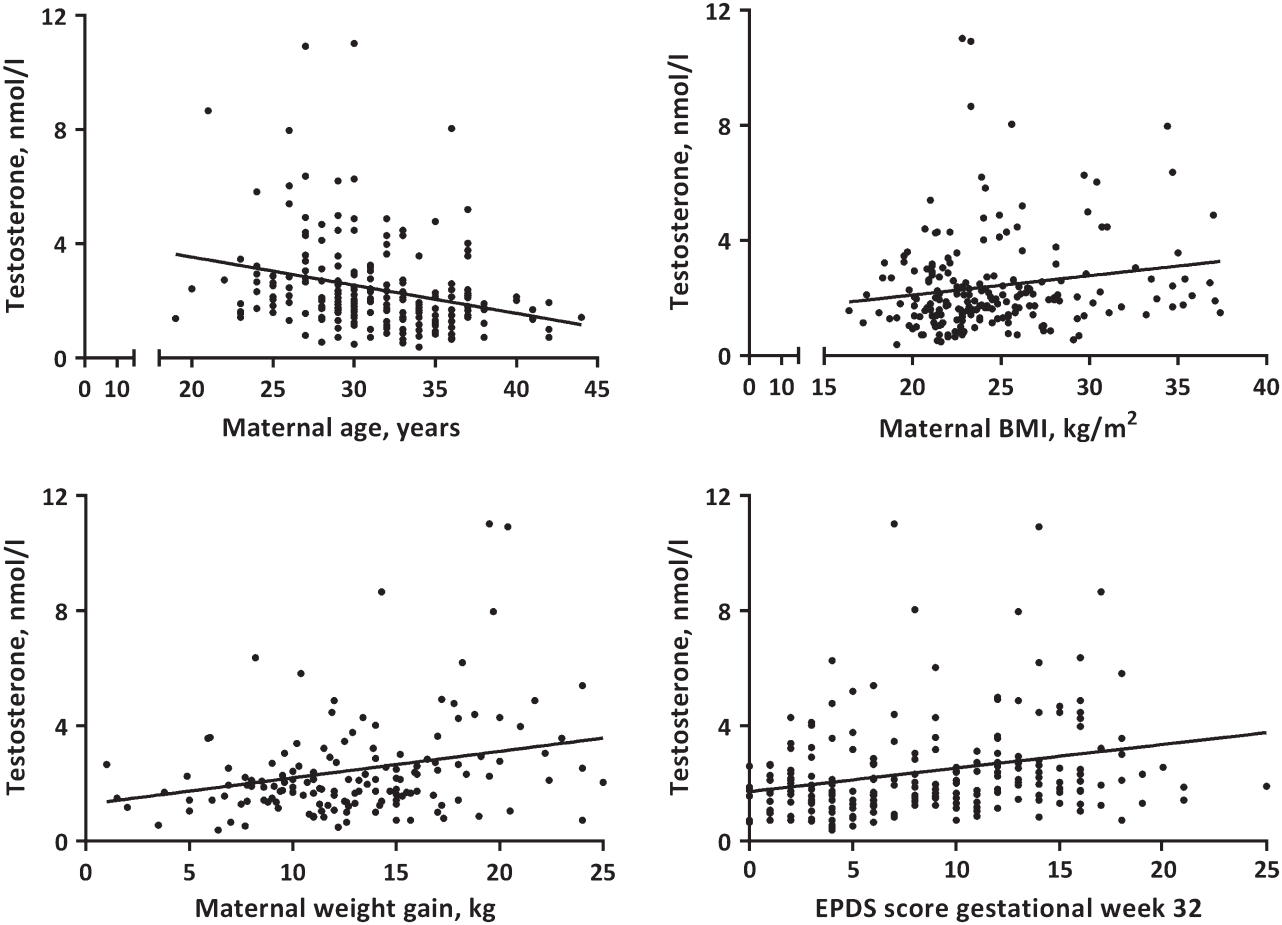
Significant Spearman rank correlations between maternal total testosterone levels in late pregnancy and maternal age, BMI, weight gain and self-rated depression scores.

Maternal total and bioavailable testosterone were significantly negatively correlated with maternal age, and positively correlated with maternal BMI at first antenatal visit, gestational weight gain and self-rated depression scores in gestational week 17 and 32 (Fig. 1 and Table 2). Primiparous women had higher total and bioavailable testosterone in comparison with multiparous women (Supplementary Table 2).

**Table 2:** Spearman rank correlation coefficients for the unadjusted association between maternal and amniotic fluid testosterone levels and maternal and fetal factors.

		**Study population I maternal serum testosterone**				**Study population II female fetus amniotic fluid testosterone**		
		**Total testosterone**		**Bioavailable testosterone**		**Total testosterone**		
**Variable**	*n*	**Spearman’s Rho**	***P***	**Spearman’s Rho**	***P***	*n*	**Spearman’s Rho**	***P***
Maternal age	216	−0.31	0.001	−0.25	0.001	56	−0.53	0.001
Maternal BMI first antenatal visit	210	0.17	0.017	0.24	0001	56	0.22	0.1
Maternal weight gain last visit	150	0.25	0.001	0.24	0.003	50	0.19	0.2
Gestational age	216	0.05	0.4	0.06	0.4	56	0.32	0.018
EPDS score gestational week 17	207	0.23	0.001	0.18	0.008	56	0.16	0.3
EPDS score gestational week 32	213	0.27	0.001	0.20	0.004	54	0.02	0.9
Amniotic fluid cortisol		–		–		56	0.56	0.001

EPDS, Edinburgh Postnatal Depression Scale.

In the multivariable linear regression models, maternal age and multiparity were independent negative explanatory variables of total testosterone, whereas BMI and self-rated depressive symptoms were independent positive explanatory variables (Table 3). Addition of PCOS diagnosis to the model did not change the estimates. When weight gain was introduced in the model, multiparity, self-rated depression and weight gain remained significant, independent explanatory factors for the maternal total testosterone levels, Table 3. Overall, these two models explained 17.8% and 26.4% of the variability in maternal total testosterone, respectively, Table 3.

**Table 3:** Linear regressions of natural logarithm transformed total maternal testosterone levels with possible predictors with significant bivariate associations.

**Covariate**	**Unstandardized** ***β*** (95% CI)	**Standardized** ***β***	***P***	**R^2^**
Model 1				*0.178*
First trimester BMI	0.02 (0.0–0.04)	0.15	0.020	
Age	−0.03 (−0.04 to −0.01)	−0.19	0.008	
Multiparity	−0.20 (−0.37 to −0.04)	−0.17	0.016	
EPDS score, week 32	0.02 (0.01–0.04)	0.21	0.001	
Model 2				*0.264*
First trimester BMI	0.02 (−0.00 to 0.04)	0.12	0.105	
Age	−0.01 (−0.03 to 0.01)	−0.08	0.365	
Multiparity	−0.34 (−0.54 to −0.15)	−0.28	<0.001	
EPDS score, week 32	0.03 (0.01–0.05)	0.26	<0.001	
Gestational weight gain	0.02 (0.00–0.04)	0.18	0.020	

### Maternal testosterone is influenced by a single nucleotide polymorphism in the aromatase gene

Genotyping analysis of the aromatase (*CYP19A1)* gene in maternal blood DNA showed that rs700518 had borderline significant effect on maternal total testosterone levels (U = 5.2; *P* = 0.073). When considering the sex of the child, a similar pattern on total testosterone levels was observed in mothers of male children *only*, where there was a significant effect by rs700815 genotype on total testosterone levels (U = 7.3, *P* = 0.026). Mothers of male children, carrying the CC genotype, had higher levels than heterozygous CT genotype (Z = 2.6, *P* = 0.009, Table 4), and also when compared with all mothers of male children carrying the T allele (CC genotype: 2.67 nmol/L (IQR: 1.96–3.24) vs t-allele carriers 1.79 nmol/L (IQR: 1.37–2.75), Z = 2.3, *P* = 0.02). None of the four other SNPs within the aromatase gene were associated with testosterone levels, data not shown.

**Table 4:** Maternal total testosterone levels (median and interquartile range) in relation to fetal sex and the rs700518 genotype of the *aromatase* gene (*n* = 199).

	**Male fetus**			**Female fetus**		
	**TT**	**TC**	**CC**	**TT**	**TC**	**CC**
*n* (%)	32 (30.2)	50 (47.2)	24 (22.6)	25 (26.9)	52 (55.9)	16 (17.2)
Maternal testosterone (nmol)	2.01 (1.45–2.93)	1.74 (1.14–2.53)	2.67 (1.96–3.24)^a^	2.02 (1.31–3.24)	1.79 (1.43–2.49)	2.16 (1.27–2.65)

a Pregnant women carrying the rs700518 CC genotype had significantly higher testosterone levels than TC genotype carriers, but only if they were expecting a male offspring, *P* = 0.009, Kruskal–Wallis test, followed by Mann–Whitney *U* test.

### Female fetus amniotic fluid testosterone is influenced by age, weight gain and cortisol levels

**Figure 2:**
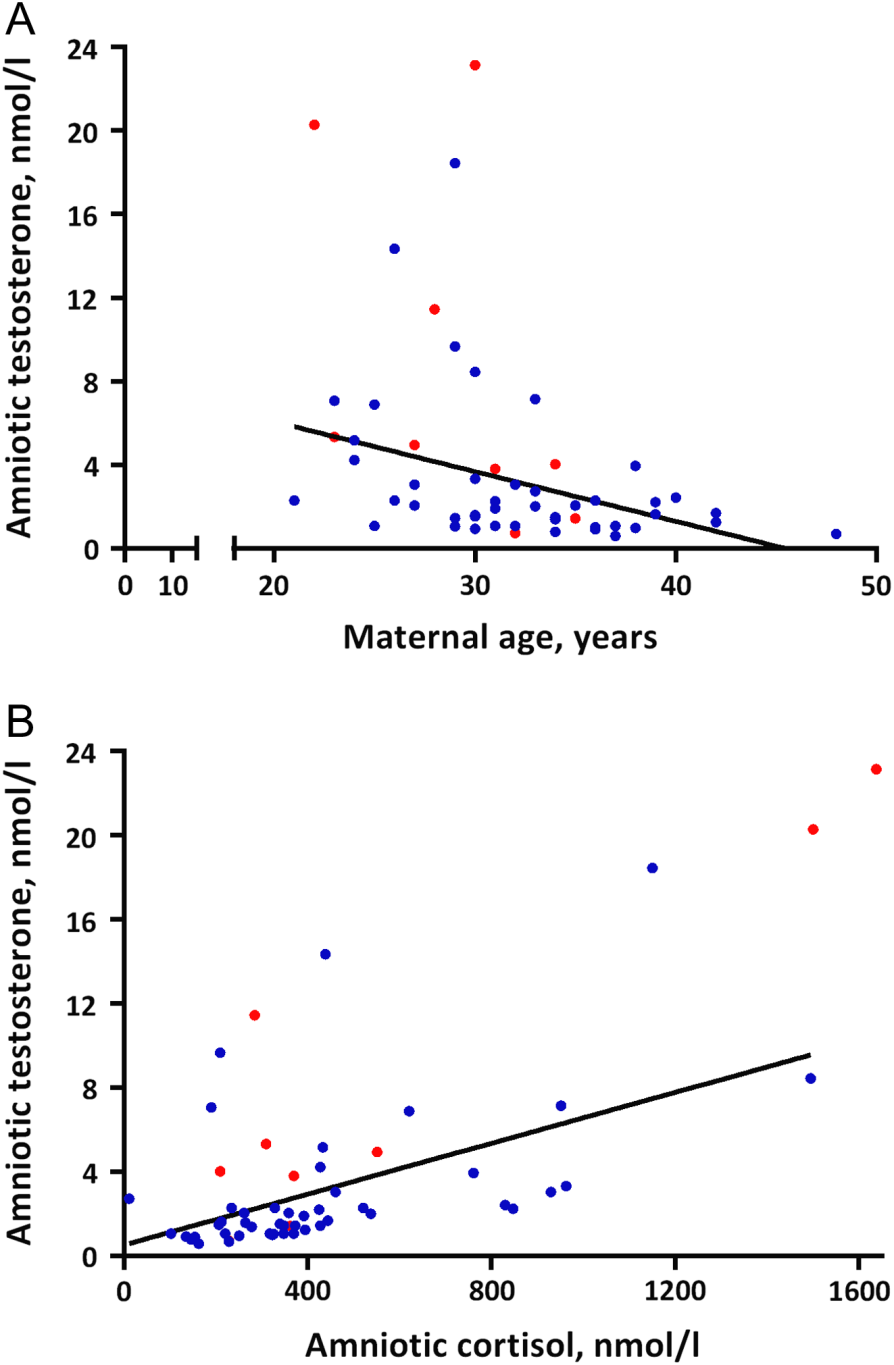
Significant Spearman rank correlations between amniotic fluid testosterone levels of female fetuses in late pregnancy and (A) maternal age and (B) amniotic fluid cortisol levels. Red dots indicate a maternal weight gain of more than one standard deviation score from the cohort mean (>18.9 kg), blue dots indicate a maternal weight gain less than one standard deviation score.

According to the bivariate analyses, amniotic fluid testosterone was significantly negatively correlated with maternal age but positively correlated with gestational age and cortisol levels (Fig. 2 and Table 2). Similarly, amniotic fluid testosterone was higher in primiparas than in multiparas and lower in women who had undergone assisted reproduction (Supplementary Table 2). In addition, the amniotic fluid testosterone was higher in women with a weight gain exceeding one S.D. from the cohort mean (4.96 nmol/L (IQR: 2.62–15.9) vs 1.96 nmol/L (IQR: 1.07–3.12), *P* < 0.05, Supplementary Table 2).

In the multivariable linear regression model maternal age, maternal weight gain and amniotic fluid cortisol levels remained independent explanatory variables of amniotic fluid testosterone, explaining 64.3% of the variability (Table 5). The significant predictors remained when cortisol was excluded from the model (data not shown). The relationship with assisted reproduction was no longer evident following adjustment.

**Table 5:** Linear regressions of natural logarithm transformed total amniotic fluid testosterone levels with possible predictors based on significant bivariate associations.

**Covariate**	**Unstandardized** ***β*** (95% CI)	**Standardized** ***β***	***P***	**R^2^**
				*0.643*
First trimester BMI	0.02 (−0.01 to 0.06)	0.12	0.2	
Age	−0.06 (−0.09 to −0.03)	−0.34	0.001	
Weight gain > 1 s.d. above mean	0.47 (0.02–0.92)	0.19	0.041	
Parity	−0.23 (−0.59 to 0.14)	−0.12	0.3	
Gestational length	0.00 (−0.02 to 0.02)	0.02	0.9	
PCOS	−0.46 (−1.35 to 0.41)	−0.10	0.3	
Assisted reproduction	−0.45 (−1.01 to 0.12)	−0.15	0.2	
Cortisol, s.d.	0.47 (0.26–0.68)	0.44	0.001	

## Discussion

The present study suggests that maternal weight gain, parity and depressed mood influence total testosterone levels in pregnant women. Additionally, in mothers carrying a male fetus, the rs700518 genotype for the aromatase enzyme had a significant effect on maternal testosterone levels. Finally, this study identified that a significant part of the variance in amniotic fluid testosterone levels of female fetuses can be explained by maternal age, maternal weight gain and fetal cortisol levels.

The explanatory variables of maternal testosterone levels are in line with previous studies (11, 12, 13), but gestational weight gain emerges as a new and relevant predictor. In non-pregnant women, 25% of circulating testosterone is derived from the adrenals, 25% from the ovary and the remaining 50% from the peripheral conversion of androstenedione (36). Enzymes needed for conversion to testosterone, 3β-hydroxy steroid dehydrogenase and 17β-hydroxy steroid dehydrogenase, are both present in the liver and adipose tissue (36). The sources for testosterone synthesis during pregnancy include the feto–placental unit, the maternal ovaries (at least during early pregnancy), the adrenals and potentially the adipose tissue also (8). From our results it is difficult to delineate which biological mechanisms mediate the associations with testosterone. However, it was recently demonstrated that high BMI interferes with the initial steps of placental cholesterol metabolism, leading to lower estradiol and progesterone levels (37). Thus, it seems reasonable to assume that the positive association between weight gain (and BMI) and testosterone levels are not driven by increased placental conversion of dihydroepiandrostenedione (DHEA) or placental *de novo* synthesis of testosterone. Meanwhile, as women with PCOS have higher testosterone levels during pregnancy, the complete suppression of ovarian activity during pregnancy may be questioned (8, 9). Further, the relationship between weight gain and maternal testosterone levels may also be due to increased androgen conversion in adipose tissue. Regardless of the mechanism, pregnancy seems to have a large impact, as the association between weight gain and total testosterone has been reported to be much weaker in non-pregnant women (38). Further, the negative associations with maternal age and parity have been noted previously (11, 12). Also in non-pregnant women, both total and free testosterone are clearly decreasing with age (39). However, the impact of parity seen in our study is substantially higher than that reported in non-pregnant populations (38). Thus, the age-effect, as well as the effect of parity, is most likely explained by age-induced changes in ovarian testosterone synthesis (40). Total maternal serum testosterone has previously been observed to be positively correlated with variables associated with increased allostatic load such as smoking, minority status, preeclampsia and high BMI (10, 13). To this list, the present study adds an association between testosterone levels and depressive symptoms. While women with PCOS more commonly suffer from anxiety and depression, and especially so, if they have high levels of free testosterone (41), there is no proven causality for testosterone in the development and maintenance of depression in women. Furthermore, in the general population, depressive symptoms do not seem to be associated with increased testosterone in women (42). We can only speculate about the positive relationship in our pregnant sample. One possibility may be that increased psychological stress, increases adrenal androgen synthesis during pregnancy.

Inter-individual differences in testosterone levels are, in part, determined by common genetic variations. Mothers carrying a male fetus, homozygous for the rs700518 C allele, had higher testosterone levels than the heterozygous CT genotype, and also higher testosterone compared with mothers carrying the T allele. This SNP has been consistently associated with estradiol serum levels, mainly in men (20, 21, 22); men homozygous for the T allele have higher estradiol serum levels, and display lower circulating testosterone levels, and higher estradiol-to-testosterone ratio (22). Therefore, the CC genotype would presumably be associated with lower aromatase enzyme activity, and in turn, with testosterone-related conditions. Despite being a synonymous polymorphism, the location of this SNP on an exonic region, and its associations with sex steroid hormones levels, would speak in favor of its (or a linked SNP) functionality. Potentially, our finding suggests that the mechanisms normally capable of restricting testosterone exposure across the placenta may be challenged in some circumstances.

Almost 65% of the amniotic fluid testosterone levels of female fetuses were explained by maternal age, maternal weight gain and fetal cortisol levels. Only one previous attempt has been made to link maternal weight gain with fetal androgen and estrogen levels (14). However, the study by Faupel-Badger *et al.* used mixed venous and arterial umbilical cord serum collected at delivery, i.e. reflecting both placental and fetal sources and did not control for cortisol levels (14). It is generally assumed that placental aromatase is an effective barrier against maternal androgens (18). In support of this, several studies have been unable to correlate maternal testosterone levels with umbilical cord levels of testosterone (15, 16, 17) or amniotic fluid with umbilical cord levels of testosterone (17). In addition, findings in women with polycystic ovary syndrome, who have high testosterone levels in pregnancy, are also conflicting, with unchanged or increased umbilical cord testosterone levels reported in the female offspring (8, 43). However, being born is stressful, and fetal cortisol, adrenaline and noradrenaline levels are increased following vaginal delivery (32). Given the strong correlation between amniotic fluid cortisol and testosterone, noted by us and others (27), and the stress induced by labor and delivery, the discrepancies in prior studies could simply be due to delivery-induced adrenal activation, rendering umbilical cord samples non-representative of the fetal exposure to testosterone throughout pregnancy. Clearly, this study is unique as amniotic fluid samples were obtained in late pregnancy, but before the onset of labor. At this stage, the fetal cortisol levels can be assumed to reflect fetal maturity, but potentially also varying degrees of fetal stress or distress. With the use of pre-labor amniotic fluid samples, we were able to demonstrate that variables that influence maternal total testosterone levels are also of relevance for the female fetus testosterone exposure.

There are many limitations to this cross-sectional study. First, the amniotic fluid study is based on a convenience sample. It is apparent that the number of women with excessive weight gain was limited, which increase the chances of type I errors. Second, we lack maternal blood samples taken at the same time as the amniotic fluid samples, thus missing the opportunity to partially correlate maternal and fetal testosterone levels, with control for fetal cortisol excretion. Finally, our calculated bioavailable testosterone levels are likely underestimating the truly bioavailable levels. High levels of estrogens during pregnancy leaves fewer SHBG-binding sites for testosterone, but since we did not obtain estrone or dihydrotestosterone levels, we could not readily employ the multi-ligand model suggested by Mazer (34).

In conclusion, parity, self-rated depression and pregnancy weight gain were associated with maternal total testosterone levels during pregnancy. With the testosterone load of a male fetus, a specific SNP in the aromatase gene will also contribute to the maternal testosterone levels. Importantly, maternal age, weight gain and fetal stress will also contribute to the testosterone exposure of female fetuses.

## Supplementary Material

Supporting Table 1

Supporting Table 2

## Declaration of interest

The authors declare that there is no conflict of interest that could be perceived as prejudicing the impartiality of the research reported.

## Funding

Fredrik och Ingrid Thurings stiftelse (CH), The Medical Faculty of Uppsala University (CH), Swedish Research Council; 2015-4870 (JB), 2013-2339 (ISP), 523-2014-2342 (AS), 2015-00495 (EC) Marianne and Marcus Wallenberg Foundation (AS), The county council of Sörmland (OA), Marta Lundqvist Foundation (2013, 2014, EC) and Swedish Society of Medicine (SLS-331991, EC). E.C. is a Marie Skłodowska Curie fellow and received funds called the EU FP7-People-Cofund (INCA 600398).

## Author contribution statement

I S P, T K-K, A S, O A, C H and R C were involved in the conception and design of the study. I S P, T K-K, A S and C H participated in the acquisition of data. K U, J B, E C, L S-F, I S P, T K-K and C H did the data analyses and R C, A S and O A contributed to the interpretation. I S P, T K-K, L S-F and C H drafted the article, and all authors revised the manuscript critically for important intellectual content. All authors have approved the final version of the manuscript.
