# Supplementary material for: Maternal and female fetal testosterone levels are associated with maternal age and gestational weight gain
Source: Eur J Endocrinol. 2017 Jul 11;177(4):379–88. doi: 10.1530/EJE-17-0207 (PMC5597951; doi:10.1530/EJE-17-0207)
Supplement: Supporting Table 1 [file eje-177-379-t001.pdf]

Supplementary Table 1. Genotype and allele frequencies if aromatase (*CYP19A1)* rs28757184, rs56658716, rs2236722, rs700518, rs6493497 single nucleotide polymorphisms.

**Marker**	**rs6493497**	**rs2236722**	**rs700518**	**rs28757184**	**rs56658716**
	[A/G]	[C/T]	[C/T]	[C/T]	[C/T]
Chromosome	15:51338638	15:51242798	15:51236915	15:51222375	15:51212492
Call Rate	0.93	0.93	0.92	0.93	0.93
Number of Alleles	2	1	2	2	1
Minor Allele (D)	A		C	T	
Minor Allele Frequency	0.09	0	0.46	0.017	0
Major Allele (d)	G	A	T	C	A
Major Allele Frequency	0.90	1	0.54	0.98	1
Fisher's HWE P	0.23	1	0.67	1	1
Genotype DD Count	0	0	40	0	0
Genotype Dd Count	38	0	103	7	0
Genotype dd Count	163	201	56	194	200
Missing Genotype Count	14	14	16	14	15
