# Supplementary material for: Maternal and female fetal testosterone levels are associated with maternal age and gestational weight gain
Source: Eur J Endocrinol. 2017 Jul 11;177(4):379–88. doi: 10.1530/EJE-17-0207 (PMC5597951; doi:10.1530/EJE-17-0207)
Supplement: Supporting Table 2 [file eje-177-379-t002.pdf]

Supplementary Table 2. Group comparisons of maternal total testosterone levels, maternal bioavailable testosterone (A) and amniotic fluid testosterone (B) across categorical variables with statistical significances according to Mann-Whitney *U* tests.

A. Maternal testosterone

	**Total testosterone nmol/l**	**p**	**Bioavailable testosterone**	**p**
**Parity**		0.001		0.002
Primipara (n = 97)	2.12 (1.61– 3.58)		0.08 (0.05 – 0.14)	
Multipara (n = 118)	1.73 (1.27 –2.40)		0.06 (0.04 – 0.09)	
**Sex of child**		0.6		0.5
Female (n = 96)	1.94 (1.36 – 2.57)		0.07 (0.04 - 0.11)	
Male (n = 117)	1.95 (1.41 – 2.92)		0.07 (0.05 - 0.12)	
**Smoking**		0.6		0.4
Yes (n = 8)	1.87 (1.43 – 2.11)		0.06 (0.04 – 0.09)	
No (n = 208)	1.95 (1.40 – 2.79)		0.07 (0.04 – 0.12)	
**Polycystic ovary syndrome**		0.6		0.8
Yes (n = 5)	2.62 (1.05 – 5.61)		0.06 (0.04 – 0.26)	
No (n = 210)	1.94 (1.40 – 2.73)		0.07 (0.04 – 0.12)	
**Assisted reproduction**		0.4		0.9
Yes (n = 12)	2.32 (1.10 – 3.48)		0.06 (0.04 – 1.00)	
No (n = 204)	1.94 (1.41 – 2.74)		0.07 (0.04 – 0.12)	
**Gestational hypertension**		0.6		0.3

Yes (n = 8)	2.08 (1.69 – 3.01)	0.08 (0.06 – 0.13)
No (n = 208)	1.94 (1.40 – 2.72)	0.07 (0.04 – 0.12)

B. Amniotic fluid testosterone

	Total testosterone nmol/l	*p*
**Parity**		0.005
Primipara (n = 21)	3.33 (1.75 – 10.6)	
Multipara (n = 35)	1.59 (1.07 –3.05)	
**Smoking**		0.3
Yes (n = 5)	2.29 (2.25 – 5.06)	
No (n = 51)	1.91 (1.07 – 4.23)	
**Weight gain**		0.031
>1 standard deviation (n = 9)	4.96 (2.62 – 15.9)	
<1 standard deviation (n = 46)	1.96 (1.07 – 3.12)	
**Polycystic ovary syndrome**		1.0
Yes (n = 2)	3.02	
No (n = 54)	2.05 (1.20 – 4.07)	
**Assisted reproduction**		0.007
Yes (n = 7)	1.07 (0.94 – 1.46)	
No (n = 49)	2.29 (1.42 – 5.06)	
**Gestational hypertension**		0.9
Yes (n = 4)	2.29 (1.09 – 3.59)	
No (n = 52)	2.03 (1.12 – 4.78)	
